# Influence of microbiota and metabolites on the quality of tobacco during fermentation

**DOI:** 10.1186/s12866-020-02035-8

**Published:** 2020-11-19

**Authors:** Jingjing Li, Yuanyuan Zhao, Yanqing Qin, Hongzhi Shi

**Affiliations:** 1 College of Tobacco Science, Henan Agricultural University, No. 95 Wenhua Road, Zhengzhou, 450002 Henan Province China; 2 Sichuan Tobacco Company, Chengdu, Sichuan China

**Keywords:** Tobacco fermentation, Temperature, Time, Microbiota, Metabolites

## Abstract

**Background:**

To explore the optimum fermentation conditions for tobacco leaves and also screen the microbiota and metabolites that are beneficial for fermentation.

**Methods:**

Tobacco leaves were fermented at 25 °C, 35 °C, and 45 °C for 2, 4, and 6 weeks, respectively. For identification of the best fermentation temperature, physicochemical properties and sensory quality of fermented tobacco were investigated. Subsequently, based on the appropriate temperature, 16 s rRNA sequencing and metabolomics analysis of tobacco were performed to monitor the change of microbes and metabolites during fermentation process (from 2 to 6 weeks).

**Results:**

Sensory quality analysis indicated that fermentation at 45 °C for 6 weeks represented the optimum condition. Metabolomics analysis showed that a total of 415 metabolites were annotated. The increase of fermentation period led to significant changes of metabolites. Results revealed an increase in concentration of L-phenylalanine and sphingosine as well as decreased concentration of betaine and phytosphingosine with the prolongation of fermentation period (2 to 6 weeks). Distinct changes in the microbiota were also observed with prolongation of the fermentation time. Results revealed that *Pseudomonas*, *Pantoea*, and *Burkholderia* were dominant bacteria in fermentation at 45 °C for 6 weeks. With the extension of the fermentation time, the abundance of *Pseudomonas* increased, while that of *Sphingomonas* and *Methylobacterium* decreased. Furthermore, microbiota profiles were tightly relevant to the altered metabolites, especially compounds involved in the sphingolipid metabolism.

**Conclusion:**

Suitable fermentation conditions were 45 °C for 6 weeks; phytosphingosine and sphingosine might affect tobacco fermentation via the sphingolipid metabolism pathway. This study provides a theoretical basis for guiding tobacco fermentation and gives insights into reducing harmful substances during tobacco fermentation.

**Supplementary Information:**

The online version contains supplementary material available at 10.1186/s12866-020-02035-8.

## Background

Tobacco (*Nicotiana tabacum* L.) is one of the most economically important non-food cultivated products in the world [1]. China is the largest producer and consumer of tobacco worldwide, according for approximately one-third of total global consumption each year. Statistically, 315 million smokers in China consume 44% of the world’s cigarettes [2]. To our knowledge, fermentation, also known as aging, is an essential process for improving the qualities of tobacco [3]. The unaged tobacco leaves cannot be directly utilized in cigarette production due to their insufficient fragrance as well as irritating smoke [4]. The fermentation process causes the tobacco to have high commercial quality and turns its color to dark yellow, eliminating harmful odors, degrading harmful substances, reducing offensive odor, and producing tobacco-specific flavors [5, 6].

Stacked fermentation is a common type of fermentation [7]. Under the condition of certain moisture content, tobacco leaves are stacked into a pile. Relying on the heat generated by the self-heating action of tobacco, it promotes the biochemical changes in leaves and improves the quality and processing characteristics of tobacco. During this process, heat is not easily dissipated due to the poor thermal conductivity of the tobacco bulk itself, and the temperature in the stack can rise to 55–65 °C. After 5–7 days of fermentation, periodic flipping of the tobacco bulk is conducted to improve fermented tobacco homogeneity and avoid overheating. This process is repeated 2–5 times and the fermentation is basically over [8]. Generally, the common artificial fermentation temperatures range from 35 to 50 °C with a fermentation cycle of 4–7 weeks [9]. However, due to the different producing area, year, variety, grade, and maturity of tobacco leaves, the optimum fermentation conditions of tobacco are various [10]. For instance, Yang et al. [11] found that Leshan Jiajiang tobacco (a type of cigar, GQH-J1) was suitable for fermentation at a temperature of 45 °C and a relative humidity (RH) of 85% for 40 days. Our previous study suggested that the optimum conditions for cigar filler tobacco fermentation were 45 °C and RH 80% [12]. Furthermore, undesirable fermentation by-products are produced during the fermentation process, such as tobacco-specific nitrosamines (TSNAs), whose increase in concentration parallels that of nitrite [13]. Another unpleasant by-product of organic acid metabolism is oxalic acid, which can negatively affect the taste of tobacco [14]. Notably, TSNAs play a central role in tobacco-smoke mediated cancer initiation [15]. Taken together, minimizing the production of TSNAs and nitrite is a major goal of tobacco fermentation technology [16].

Reportedly, fermentation not only is a chemical reaction process, but also is linked to the enzymatic action of microbes, which play extremely vital roles in this process [17]. Di Giacomo et al. [18] revealed that *Staphylococcaceae* and *Lactobacillales* were dominant detected bacteria in the early fermentation, whereas *Actinomycetales* rapidly expanded during the late phase. Meanwhile, several microorganism strains, such as AS97 (a kind of *Pseudomonas fluorescens*, Genbank accession number: JF449445) [19] and cellulose degradation bacteria [20], have the ability to reduce TSNAs in cigarettes, which can potentially be applied for industrial tobacco fermentation. Besides, changes in chemical compositions and metabolites in the tobacco fermentation process have been found [21, 22]. Previous study indicated that the organic acid content, volatile acid, and volatile carbonyl compounds were increased, while total nitrogen, nicotine, protein, and amino acids were decreased during the fermentation process [5]. Besides, polyphenols are converted into aroma substances via oxidative degradation, which improves the aroma of cigarette [23]. Despite the studies mentioned above, few studies have systematically investigated the changes of microbiota composition and metabolites during the tobacco fermentation. Thus, in order to improve the quality of the end product and possibly improve its safety, it is necessary to determine the type of microorganisms and metabolites that play roles in this process and to identify the optimal fermentation conditions.

In the present study, we selected Shiyan No.1 as the research material to investigate its optimal fermentation condition and explore changes in microbiota and metabolites during fermentation. Concretely, the tobacco leaves were fermented at 25 °C, 35 °C, and 45 °C for 2, 4, and 6 weeks, respectively. According to the physiological indicators and sensory evaluation of fermented tobacco leaves, the optimal conditions of tobacco fermentation were obtained. Then, based on optimal temperature, we identified the altered microorganisms and metabolites during the fermentation process (2, 4 and 6 weeks) by using 16 s rRNA gene sequencing and liquid chromatography-mass spectrometry (LC-MS) analysis.

## Results

### Sensory quality assessment

The content of the aroma components is listed in Table 1. The results showed that the levels of major neutral aroma components, such as solanone, geranylacetone, dihydroactinidiolide, and megastigmatrienone, were elevated with the increase in fermenting temperature. These components played vital roles in producing fragrances and increasing the concentration of tobacco smoke. In addition, the neophytadiene content was the highest at 45 °C compared with the others. The neophytadiene could enhance aroma, reduce irritants and alcohol, and was considered as one of the key ingredients that contributed the most to the aroma of tobacco. Thus, 45 °C fermentation was the most conducive to the accumulation of aroma components in tobacco leaves.

**Table 1:** The content of aroma components of tobacco leaves in different groups

Components (μg/g)	Groups									
	control	25 °C/2 weeks	25 °C/4 weeks	25 °C/6 weeks	35 °C/2 weeks	35 °C/4 weeks	35 °C/6 weeks	45 °C/2 weeks	45 °C/4 weeks	45 °C/6 weeks
Solanone	4.758	4.938	5.208	6.655	7.194	7.447	7.476	8.439	12.002	12.008
Geranylacetone	0.500	0.530	0.593	1.171	0.946	1.209	1.344	1.373	1.753	1.908
β- ionone	0.902	0.877	0.840	0.752	0.808	0.782	0.685	0.801	0.761	0.603
Ionone oxide	2.789	2.500	2.190	2.071	2.299	2.102	1.755	2.188	1.953	1.422
Dihydroactinidiolide	4.224	4.699	5.247	6.565	5.868	7.340	8.350	7.008	7.608	8.215
Megastigmatrienone 1	0.465	0.503	0.538	0.555	0.617	0.709	0.818	0.829	0.984	1.024
Megastigmatrienone 2	3.154	3.170	3.355	3.434	3.732	4.348	4.695	4.929	5.811	6.238
Megastigmatrienone 3	0.423	0.489	0.563	0.572	0.711	0.818	0.811	0.931	1.089	1.082
3-hydroxy-β - dihydro damadone	0.236	0.256	0.305	0.487	0.750	0.890	1.067	1.178	1.644	1.700
Megastigmatrienone 4	2.068	2.104	2.111	3.963	4.471	4.911	5.037	5.434	6.076	5.689
3-Oxo-α-ionol	58.326	62.240	63.806	67.865	65.329	79.524	60.731	55.774	65.583	46.688
Neophytadiene	338.195	349.413	362.707	375.111	351.408	410.975	422.262	428.902	492.098	455.894
3-Hydroxysolavetivone	3.372	3.697	4.171	4.295	3.922	4.200	5.831	6.092	6.522	6.558
β-farnesene	7.390	8.511	11.016	12.015	9.232	13.111	12.356	11.184	14.340	12.271
Total aroma (except neophytediene)	88.607	94.511	99.945	110.400	105.879	127.391	110.956	106.159	126.128	105.408
Total aroma	426.802	443.925	462.651	485.511	457.287	538.366	533.218	535.061	618.226	561.301

The tobacco leaves were fermented at 25 °C, 35 °C, and 45 °C (90% relative humidity), and samples were collected after 2, 4, and 6 weeks

Meanwhile, with the increase of fermentation temperature and time, the content of TSNAs raised significantly (Table 2, Supplementary Figure 1A and 1B), while the level of alkaloids and nitrates decreased evidently (Table 3, Supplementary Figure 1C and 1D), suggesting that the accumulation of harmful substances in smoke declined to some extent at 45 °C fermenting. Furthermore, with the increase of temperature, the aroma quality and quantity of tobacco gradually increased, the irritation was eliminated, and the aftertaste was comfortable (Table 4). Taken together, the quality detection and sensory analysis showed that 45 °C fermenting for 6 weeks was the optimal fermentation condition.

**Table 2:** The level of NNN, NAT, NAB, NNK, and TSNAs in tobacco leaves during fermentation process

Groups	NNN (ng/g)	NAT (ng/g)	NAB (ng/g)	NNK (ng/g)	TSNAs (ng/g)
Control	812.73 ± 33.2Gg	1459.82 ± 84.60Ee	40.30 ± 0.37Hh	145.34 ± 8.07Ef	2458.19 ± 126.36Gf
25 °C/ 2 weeks	934.19 ± 31.32Ff	2060.91 ± 85.57Dd	63.81 ± 0.39Gg	242.30 ± 8.13De	3301.21 ± 125.41Fe
25 °C/ 4 weeks	1159.46 ± 33.55Ee	2274.54 ± 83.56Cc	72.49 ± 0.36Ff	293.61 ± 8.41Cd	3800.10 ± 125.88Ed
25 °C/ 6 weeks	1546.99 ± 36.10Bb	2536.65 ± 88.18ABb	84.10 ± 0.32Dd	349.86 ± 8.47ABb	4517.60 ± 133.06BCb
35 °C/ 2 weeks	976.51 ± 36.06Ff	2069.04 ± 88.04Dd	72.81 ± 0.35Ff	246.66 ± 8.22De	3365.03 ± 132.67Fe
35 °C/ 4 weeks	1251.87 ± 35.24Dd	2394.27 ± 84.81BCbc	84.34 ± 0.30Dd	300.26 ± 8.37Cd	4030.73 ± 128.72DEd
35 °C/ 6 weeks	1602.03 ± 39.96Bb	2694.59 ± 80.05Aa	90.19 ± 0.35Bb	354.84 ± 8.07ABab	4741.66 ± 128.43ABa
45 °C/ 2 weeks	1192.84 ± 33.58DEde	2294.08 ± 89.99Cc	73.86 ± 0.34Ee	287.15 ± 8.22Cd	3847.94 ± 132.12Ed
45 °C/ 4 weeks	1439.20 ± 31.50Cc	2407.34 ± 89.62BCbc	86.59 ± 0.36Cc	335.67 ± 8.30Bc	4268.80 ± 129.78CDc
45 °C/ 6 weeks	1685.79 ± 38.24Aa	2720.40 ± 84.92Aa	95.80 ± 0.34Aa	367.12 ± 8.14Aa	4869.12 ± 131.63Aa

*NNN* N′-nitrosonornicotine; *NAT* N′-nitrosoanatabine; *NAB* N′-nitrosoanabasine; *NNK* 4-(methylnitrosamino)-1 -(3-pyridyl)-1-butanone; *TSNAs* Tobacco-specific Nitrosamines. Control group represents the sun-dried unfermented tobacco leaves. All data are represented by mean ± SD (*n* = 3). Uppercase letters indicate *p*-value < 0.05 and lowercase letters indicate *p*-value < 0.01

**Table 3:** The level of alkaloids and nitrate of tobacco leaves in different groups

Groups	Nicotine (μg/g)	Nornicotine (μg/g)	Neonicotine (μg/g)	Anatabine (μg/g)	Total alkaloids (%)	Nitrate (μg/g)
Control	5.16 ± 0.07	0.24 ± 0.01	0.05 ± 0.00	0.48 ± 0.02	5.92 ± 0.10Aa	3589.33 ± 52.78Aa
25 °C/ 2 weeks	4.90 ± 0.07	0.21 ± 0.01	0.04 ± 0.00	0.41 ± 0.02	5.56 ± 0.10Bb	3354.15 ± 52.20Bb
25 °C/ 4 weeks	4.69 ± 0.08	0.19 ± 0.01	0.04 ± 0.00	0.41 ± 0.02	5.33 ± 0.10Bc	3160.14 ± 52.29Ccd
25 °C/ 6 weeks	4.24 ± 0.08	0.18 ± 0.01	0.03 ± 0.00	0.36 ± 0.02	4.82 ± 0.12CDde	2991.19 ± 54.31DEe
35 °C/ 2 weeks	4.35 ± 0.08	0.20 ± 0.01	0.03 ± 0.00	0.40 ± 0.02	4.98 ± 0.11Cd	3210.28 ± 56.96Cc
35 °C/ 4 weeks	4.25 ± 0.08	0.18 ± 0.01	0.03 ± 0.00	0.38 ± 0.02	4.85 ± 0.12CDde	2938.96 ± 55.11Ee
35 °C/ 6 weeks	4.11 ± 0.06	0.16 ± 0.01	0.03 ± 0.00	0.35 ± 0.02	4.65 ± 0.12DEef	2674.46 ± 58.02FGg
45 °C/ 2 weeks	4.31 ± 0.09	0.19 ± 0.01	0.03 ± 0.00	0.38 ± 0.02	4.91 ± 0.10CDd	3094.13 ± 51.96CDd
45 °C/ 4 weeks	4.20 ± 0.08	0.18 ± 0.01	0.03 ± 0.00	0.37 ± 0.02	4.78 ± 0.13CDde	2785.81 ± 57.53Ff
45 °C/ 6 weeks	3.96 ± 0.06	0.16 ± 0.01	0.03 ± 0.00	0.35 ± 0.02	4.50 ± 0.12Ef	2569.76 ± 53.03Gh

Control group represents the sun-dried unfermented tobacco leaves. All data are represented by mean ± SD (n = 3). Uppercase letters indicate *p*-value < 0.05 and lowercase letters indicate *p*-value < 0.01

**Table 4:** The sensory evaluation of fermented tobacco

Groups	Aroma quality 9–0	Aroma quantity 9–0	Smoke density 9–0	Offensive odor 9–0	Physiological strength 9–0	Irritation 9–0	Aftertaste 9–0	Combustibility 9–0	Cigarette ash 9–0
Control	3.5	6.0	6.0	5.0	8.2	3.0	3.5	8.0	8.0
25 °C/ 2 weeks	4.5	6.2	6.0	5.3	8.0	3.8	4.0	8.0	8.0
25 °C/ 4 weeks	5.0	6.5	6.3	5.5	7.5	4.5	5.0	8.0	8.0
25 °C/ 6 weeks	5.7	6.8	6.5	5.7	7.3	5.3	5.3	8.0	8.0
35 °C/ 2 weeks	5.0	6.3	6.2	5.5	7.5	4.5	5.0	8.0	8.0
35 °C/ 4 weeks	5.5	6.6	6.3	5.7	7.3	5.0	5.3	8.0	8.0
35 °C/ 6 weeks	5.8	7.0	6.5	5.8	7.2	5.5	5.5	8.0	8.0
45 °C/ 2 weeks	4.7	6.5	6.3	5.5	8.0	4.0	5.0	8.0	8.0
45 °C/ 4 weeks	5.5	6.8	6.5	5.8	7.5	5.2	5.3	8.0	8.0
45 °C/ 6 weeks	6.0	7.2	6.8	6.0	7.3	5.8	5.8	8.0	8.0

Aroma quality indicates the quality of aroma and higher scores represent better quality of aroma. Aroma quantity indicates the content of aroma and higher scores represent greater quantity of aroma. Smoke density indicates the concentration of aroma with higher scores representing higher concentration. Offensive odor indicates undesirable aromas produced by burning cigarettes, and higher scores represent less offensive odor. Physiological strength indicates the physical impact of smoke felt by smokers. The higher strength score means greater impact and stronger satisfaction. Irritation indicates slight and obvious sensory discomfort caused by smoke and higher scores represent less irritant gas. Aftertaste indicates the taste sensation after the smoke leaves the mouth and nasal cavity and higher scores mean greater aftertaste. Cigarette’s Combustibility refers to the combustion characteristics of tobacco products, which is an important physical characteristic of tobacco leaves, including smoldering, burning speed, burning uniformity, burning completeness, soot color as well as cohesion. Cigarette ash indicates the color of the remaining soot after burning tobacco leaves, white is the best, followed by gray

### Metabolomics analysis

#### The principal component analysis (PCA) result

The tobacco leaves fermented at 45 °C for 2 weeks (group 2), 4 weeks (group 3), and 6 weeks (group 4) as well as unfermented leaves (group 1) was selected for metabolomics analysis. The metabolites of tobacco were obtained using LC-MS analysis, and the electrospray ionization of LC-MS contained positive (POS) and negative (NEG) ion modes. Generally, PCA plot could show the trend of separation between groups in the experiment. In the present study, the PCA plots of POS and NEG revealed that samples from each group overlapped well, and there was a significant separation between group 1 and group 4 (Supplementary Figure 2). A total of 1403 and 4751 valid peaks were identified in POS and NEG modes, respectively.

#### Metabolites annotations results

**Fig. 1:**
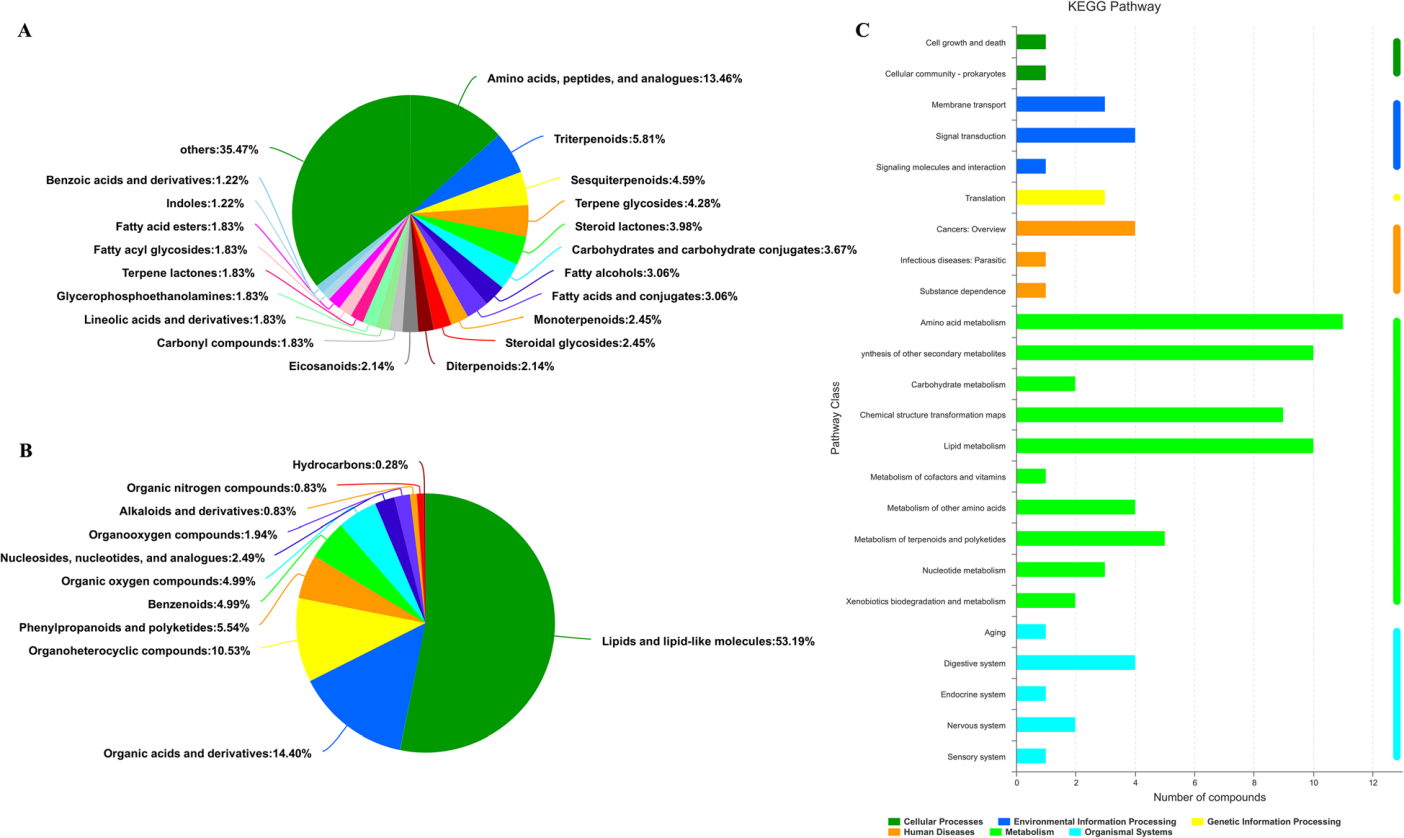
Metabolite annotations analysis of tobacco leaves from group 1 to 4. **a**: Pie-chart of the HMDB subclass; **b**: pie-chart of HMDB superclass. Each color in each pie-chart represents the different HMDB classification, and its area represents the relative proportion of metabolites in the classification. **c:** KEGG pathways of metabolites. The y-axis indicates the name of the KEGG metabolic pathway and the x-axis indicates the number of compounds that are annotated to the pathway. KEGG pathways are divided into seven categories and the color of the bars indicates the different metabolic pathway categories. HMDB: The Human Metabolome Database; KEGG: Kyoto Encyclopedia of Genes and Genomes. Group 1 represents the sun-dried unfermented tobacco leaves; group 2 represents leaves fermented at 45 °C for 2 weeks; group 3 represents leaves fermented at 45 °C for 4 weeks; and group 4 represents leaves fermented at 45 °C for 6 weeks

A total of 415 metabolites identified as mentioned above were assigned to the Kyoto Encyclopedia of Genes and Genomes (KEGG) and the Human Metabolome Database (HMDB). The detail information of metabolites is shown in Supplementary Table 1. These metabolites were classified into 93 HMDB subclasses and 11 HMDB superclasses (Fig. 1a and b). Among these, 192 metabolites were included in the “lipids and lipid-like molecules” term, which accounted for the majority of all classes (53.19%). “Organic acids and derivatives” (14.4%) and “organo-heterocyclic compounds” (10.53%) were also enriched by identified metabolites.

KEGG pathways analysis showed that 415 metabolites were divided into seven categories, including metabolism, genetic information processing, environmental information processing, cellular processes, organismal systems, human diseases, and drug development (Fig. 1c). For the “metabolism” term, the major pathways were “amino acid metabolism”, followed by “lipid metabolism”, “biosynthesis of other secondary metabolites”, and “chemical structure transformation maps”. Details of the top 20 pathways are listed in Supplementary Table 2. Notably, we found that L-phenylalanine was involved in the phenylalanine metabolism pathway (belong to amino acid metabolism); in addition, phytosphingosine and sphingosine participated in the sphingolipid metabolism pathway (belong to lipid metabolism).

#### Multivariate analysis of differential metabolites among groups

To screen the differential metabolites among these groups, we integrated the results of the multivariate analysis to identify the differential metabolites between any two groups. As shown in Supplementary Figure 3, the differential metabolites between control and fermentation groups were significantly separated based on the threshold variable importance in the projection (VIP) > 1 and *p*-value < 0.05. A total of 34 (POS) and 52 (NEG) differential metabolites were obtained between group 2 and group 1; 43 (POS) and 44 (NEG) differential metabolites were screened between group 3 and group 1; total 60 (POS) and 58 (NEG) differential metabolites were identified between group 4 and group 1. Among these, compared with the group 1, the concentrations of L-phenylalanine and sphingosine in group 4 were increased, while the concentrations of betaine and phytosphingosine were decreased.

#### Analysis of KEGG pathways

**Fig. 2:**
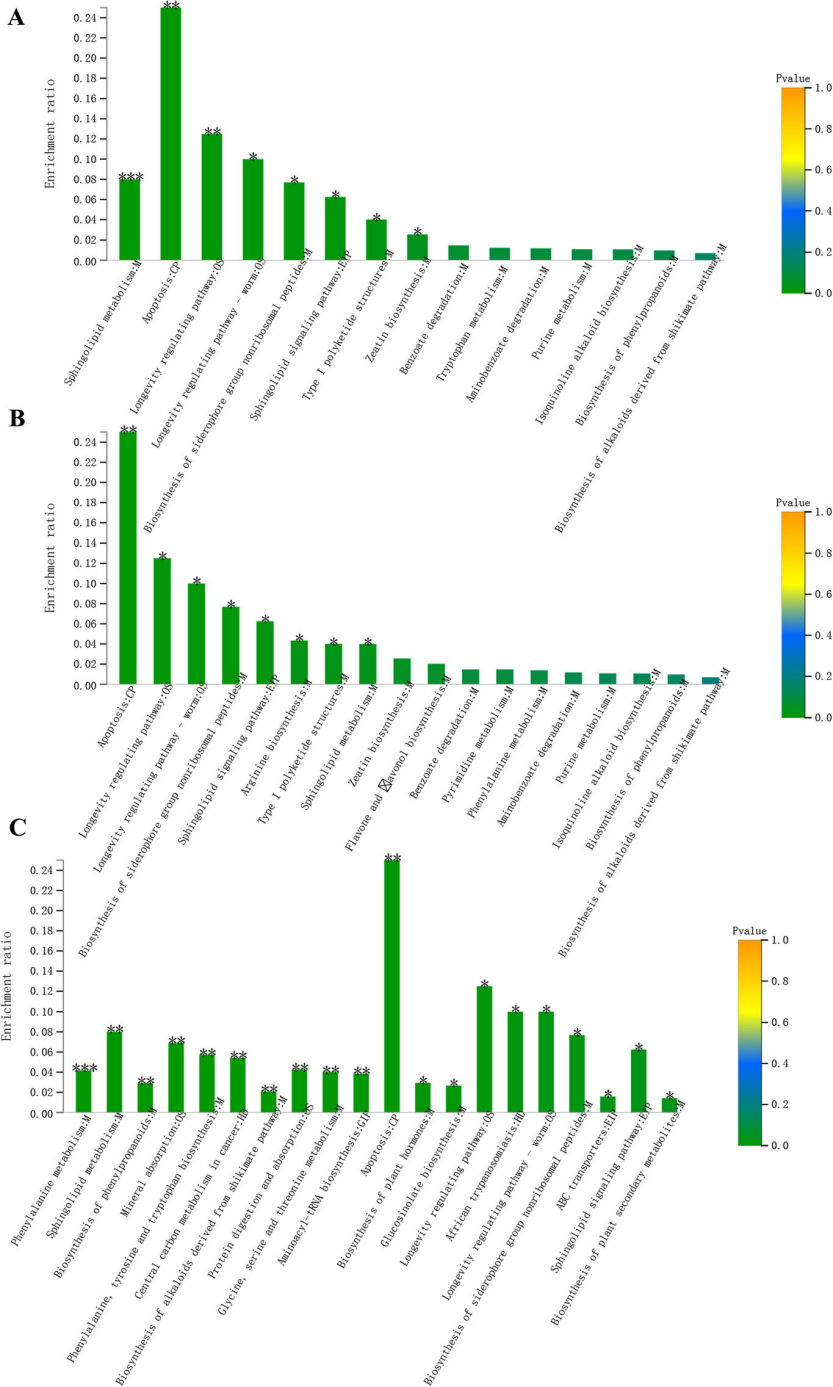
KEGG analysis of differential metabolites in comparison between the two groups. **a**: Group 2 vs. group 1; **b**: group 3 vs. group 1; **c**: group 4 vs. group 1. The x-axis represents the pathway name, and the y-axis represents the enrichment rate. *p*-value or FDR < 0.001 is marked as ***, *p*-value or FDR < 0.01 is marked as **, and *p*-value or FDR < 0.05 is marked as *. Group 1 represents the sun-dried unfermented tobacco leaves; group 2 represents leaves fermented at 45 °C for 2 weeks; group 3 represents leaves fermented at 45 °C for 4 weeks; and group 4 represents leaves fermented at 45 °C for 6 weeks

Venn analysis was performed to identify the common and specific metabolites between any two groups, and 41 metabolites were shared between these four groups. In total, 15 pathways were enriched by group 2 vs. group 1, of which eight pathways were considered significant (Fig. 2a), such as sphingolipid metabolism, apoptosis, longevity regulating pathway, and biosynthesis of siderophore group nonribosomal peptides. Additionally, 18 pathways were obtained in group 3 vs. group 1, and eight pathways were significantly enriched (Fig. 2b), which mainly included apoptosis, longevity regulating pathway, and biosynthesis of the siderophore group nonribosomal peptides. For group 4 vs. group 1, a total of 38 pathways were screened, among those, 20 pathways were found to be statistically significant (Fig. 2c). These were, for example, phenylalanine metabolism, sphingolipid metabolism, and biosynthesis of phenylpropanoids. Besides, we also observed six common pathways that were involved in these groups, including sphingolipid metabolism, apoptosis, longevity regulating pathway, longevity regulating pathway – worm, biosynthesis of siderophore group nonribosomal peptides, and sphingolipid signaling pathway.

### 16 s rDNA/rRNA amplicon sequencing analysis

#### Sequences analysis

In order to investigate the effect of microflora on tobacco fermentation, the 16 s rDNA/rRNA amplicon sequencing was conducted. A total of 2,017,972 raw reads were generated from 20 tobacco samples. After quality filtering, 1,008,986 (50%) of high-quality sequences with a mean length of 411 bp were obtained. Operational taxonomic units (OTUs) were performed based on the 97% unique sequence similarity. Thus, 1538 OTUs were detected among 20 samples.

#### Alpha-diversity analysis

**Fig. 3:**
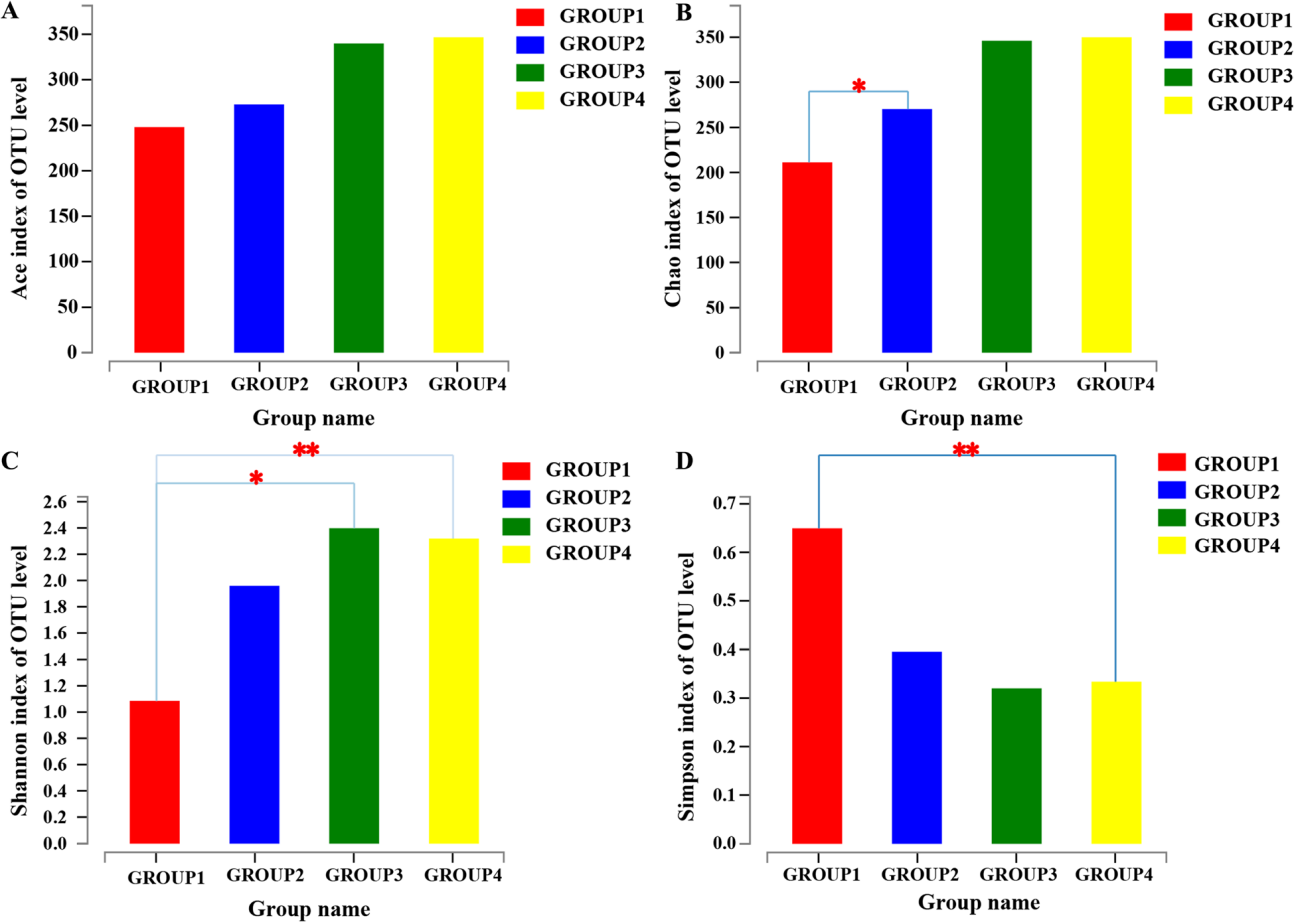
Alpha diversity comparison among four groups. **a**: Ace index; **b**: Chao index; **c**: Shannon index; **d**: Simpson index. *p*-value < 0.01 is marked as **, and *p*-value < 0.05 is marked as *. Group 1 represents the sun-dried unfermented tobacco leaves; group 2 represents leaves fermented at 45 °C for 2 weeks; group 3 represents leaves fermented at 45 °C for 4 weeks; and group 4 represents leaves fermented at 45 °C for 6 weeks

We further evaluated the bacterial diversity of all tobacco samples depending on the different fermentation time based on ace, Chao, Shannon, and Simpson indexes. The ace and Chao were used to identify the community richness, whereas Shannon and Simpson were used to evaluate community diversity. Ideally, higher Shannon value indicated higher diversity, while the higher Simpson index showed lower diversity. As shown in Fig. 3, bacterial richness and community diversity increased with prolonging fermentation time, but there was no significant difference in bacterial diversity between group 3 and 4. Additionally, Shannon and Sobs rarefaction curves for each sample are shown in Supplementary Figure 4, indicating the reasonable volume of sequencing data.

#### Taxonomic composition

**Fig. 4:**
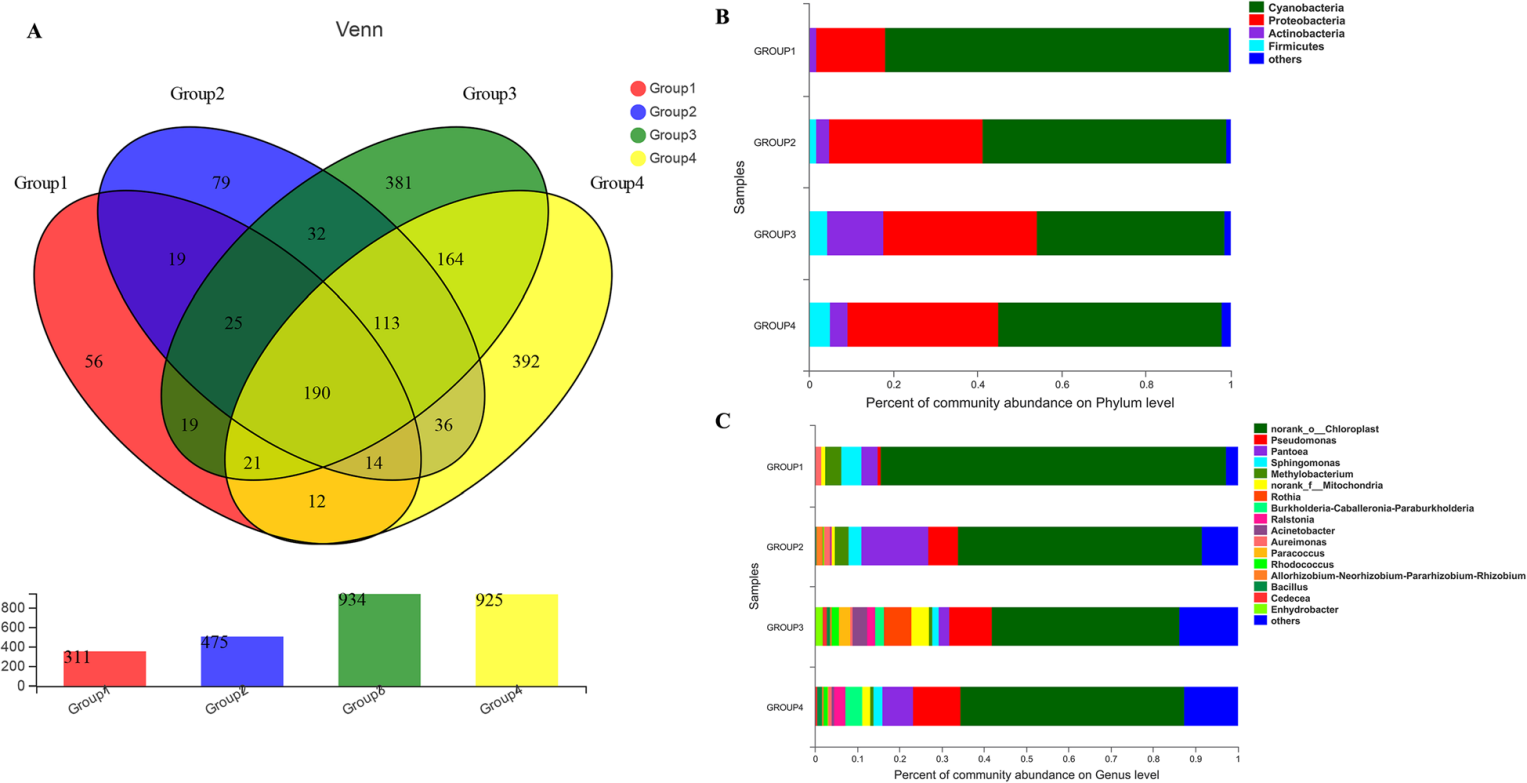
The taxonomic composition of OTUs from four groups. **a**: The number of shared and unique OTUs in different groups; **b**: Relative microbiota abundance at the phyla level; **c**: Relative microbiota abundance at the genus level. The y-axis shows the sample name, and the x-axis is the proportion of the species in the sample. The pillar of different colors represents different species, and the length of the pillar represents the proportion of the species. OTU: operational taxonomic unit. Group 1 represents the sun-dried unfermented tobacco leaves; group 2 represents leaves fermented at 45 °C for 2 weeks; group 3 represents leaves fermented at 45 °C for 4 weeks; and group 4 represents leaves fermented at 45 °C for 6 weeks

The taxonomic composition was performed using the analytical program QIIME, and the Venn diagram showed the number of shared and unique OTUs in different groups (Fig. 4a). Additionally, the sequences were classified from kingdom to species. In total, five different phyla were mainly identified among these samples (Fig. 4b). *Cyanobacteria* (80%) and *Proteobacteria* (17%) were the dominant bacteria in group 1; *Cyanobacteria*, *Proteobacteria*, and *Actinobacteria* were the prevalent bacteria in group 2, representing respectively 67%, 34%, and 5% of total sequences; *Cyanobacteria* (44%), *Proteobacteria* (40%), and *Actinobacteria* (10%) were the most frequently detected phyla in the group 3; *Cyanobacteria*, *Proteobacteria*, *Firmicutes*, and *Actinobacteria* were mainly identified bacterial strain in group 4, accounting for 46%, 34%, 7%, and 4% of all reads, respectively. We found that the abundance of *Firmicutes* increased with the prolongation of fermentation time, and change in the *Proteobacteria* population showed the same trend.

At the genus level, the raw sequences were mainly assigned into 17 different genera (Fig. 4c). In the group 1, *norank_o__Chloroplast* (82%), *Sphingomonas* (5%), *Pantoea* (4%), and *Methylobacterium* (4%) were primary genera; in the group 2, *norank_o__Chloroplast* (57%), *Pantoea* (13%), *Pseudomonas* (7%), and *Methylobacterium* (4%) were dominant bacteria; additionally, *norank_o__Chloroplast*, *Pseudomonas*, and *Rothia* were frequently identified in the group 3, representing 45%, 10%, and 7% of sequencing analysis, respectively; in the group 4, the most abundant genus were *norank_o__Chloroplast* (54%), *Pseudomonas* (11%), *Pantoea* (9%), *Burkholderia* (5%), and *Ralstonia* (4%).

#### Beta-diversity analysis

We further evaluated the impact of fermentation time on microbial tobacco structure by PCA, principal coordinate analysis (PCoA), and non-metric multidimensional scaling (NMDS) analysis (Supplementary Figure 5). In the PCA plot, the data of group 2, group 3, and group 4 were far separated from group 1, suggesting that fermentation had a significant effect on bacterial diversity in tobacco leaves. However, the PCoA and NMDS plots showed no separation among these groups.

#### The differences in community composition of samples

**Fig. 5:**
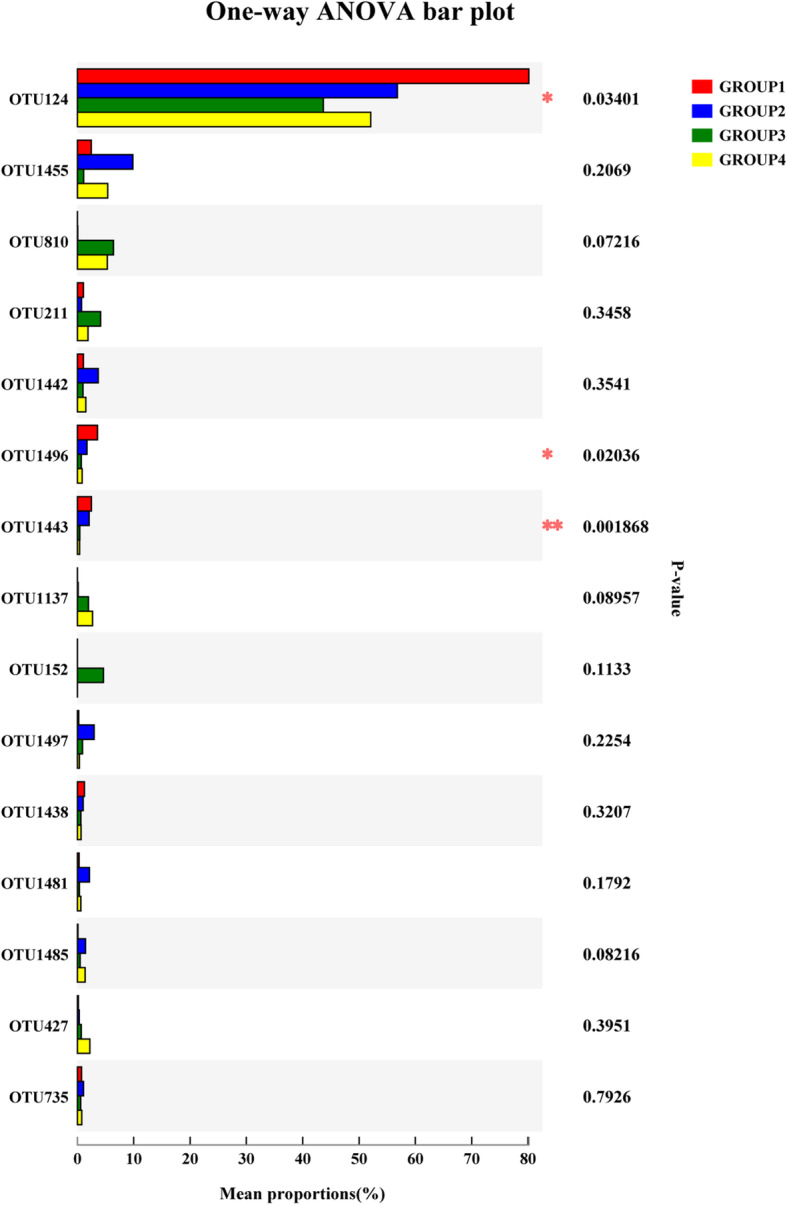
The change of differential OTUs during fermentation process. OTU124: *norank_o__Chloroplast*; OTU1455: *Pantoea*; OTU810: *Pseudomonas_brenneri*; OTU211: *Mitochondria*; OTU1442: *Pantoea*; OTU1496: *Sphingomonas*; OTU1443: *Methylobacterium*; OTU1137: *Ralstonia*; OTU152: *Rothia*; OTU1497: *Pseudomonas*; OTU1438: *Aureimonas*; OTU1481: *Pseudomonas_psychrotolerans*; OTU1485: *Pseudomonas_parafulva*; OTU427: *Pseudomonas*; OTU735: *Sphingomonas_panni.* * represents *p*-value < 0.05, ** represents *p*-value < 0.01

In order to further analyze the changes of microbiota during fermentation, we identified the differential OTUs among different groups (Fig. 5). The bar chart exhibited the 15 differential OTUs in groups; among those, three OTUs had statistically differences, including OTU124 (*norank_o__Chloroplast*), OTU1496 (*Sphingomonas*), and OTU1433 (*Methylobacterium*). The abundance of *norank_o__Chloroplast* decreased in groups 2 and 3, while it was increased in group 4. Moreover, the abundance of both *Sphingomonas* and *Methylobacterium* was reduced with the extension of fermentation time.

Furthermore, the KEGG pathway enrichment analysis of OTUs was performed. The results revealed that identified OTUs were mainly involved in the biosynthesis of ansamycins, biosynthesis of siderophore group nonribosomal peptides, and other glycan degradation and isoflavonoid biosynthesis processes (Supplementary Table 3).

### The integrated results analysis

**Fig. 6:**
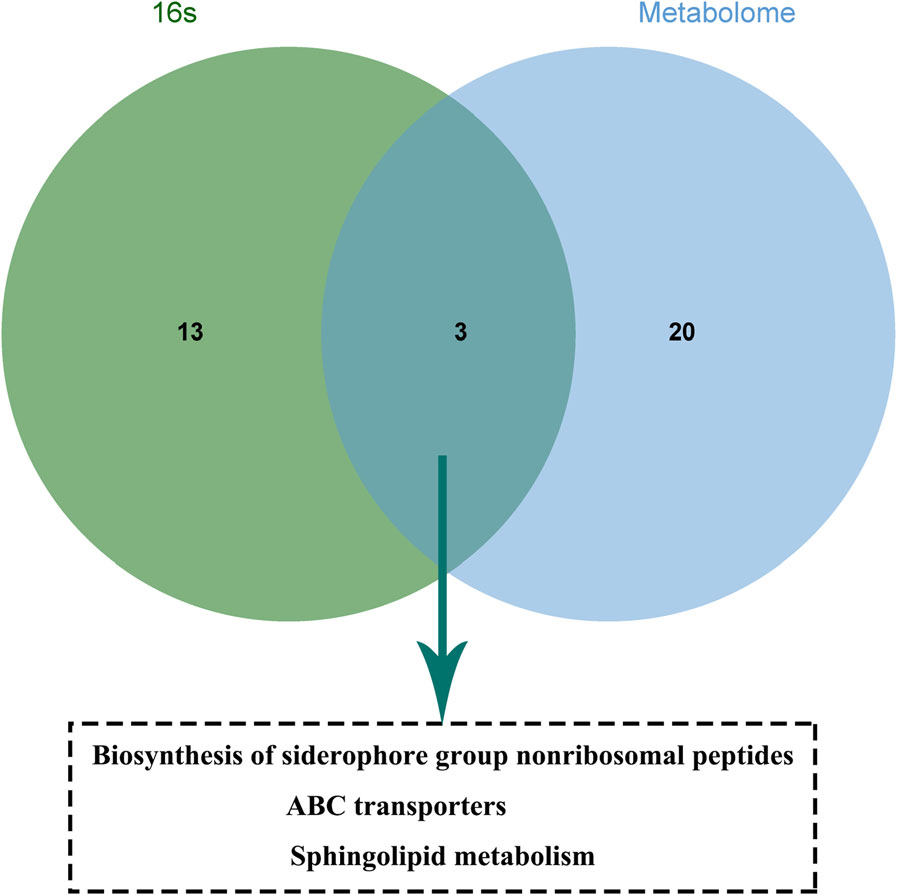
Integrated analysis of pathways enriched by microorganisms and metabolites

The pathways obtained both in 16 s rDNA/rRNA sequencing and metabolome were integrated, and the same or similar pathways were selected. A total of three pathways were identified, including biosynthesis of siderophore group nonribosomal peptides, ABC transporters, and sphingolipid metabolism (Fig. 6). In particular, 2,3-Dihydroxybenzoic acid might be involved in biosynthesis of siderophore group nonribosomal peptides; L-Histidine, L-Phenylalanine, and betaine might participate in ABC transporters; as well as phytosphingosine, sphingosine, and 3-ketosphinganine might be relevant to sphingolipid metabolism.

## Discussion

Tobacco fermentation is a crucial part of cigarette processing, which is affected by fermentation temperature and period. However, the appropriate fermentation conditions of different varieties of tobacco leaves are different. In the present study, we identified the proper fermentation conditions of Shiyan No.1, and investigated the principal metabolites as well as dominant bacteria under suitable fermentation conditions. Based on the results of physical properties, chemical compositions, and sensory quality in the fermented tobacco (Table 1, 2, 3 and 4), we detected that fermentation at 45 °C for 6 weeks was a mostly favorable condition, which was basically consistent with the commercial tobacco fermentation conditions. As for the tobacco leaves fermented in different periods at 45 °C, both the altered metabolites and OTUs were involved in three pathways, namely, biosynthesis of siderophore group nonribosomal peptides, ABC transporters, and sphingolipid metabolism (Fig. 6). Notably, the abundance of *Pseudomonas* was increased with the extension of time, while the abundances of *Sphingomonas* and *Methylobacterium* were reduced (Fig. 4). We also observed that the concentrations of L-phenylalanine and sphingosine were increased, while the concentrations of betaine and phytosphingosine were decreased in group 4 than control. Therefore, we speculated that these microflora and metabolites might play roles during the tobacco fermentation process.

In the taxonomic composition analysis, we observed that the abundance of *Pseudomonas* was elevated with the extension of fermentation time (Fig. 4). Despite nicotine plays a significant role in the cigarette properties, it is harmful to human health [24]. Thus, it is necessary to screen microbiotas with the ability to degrade nicotine. Previous studies indicated that *Pseudomonas* spp. were capable of degrading nicotine [25, 26]. Yu et al. [27] also observed that *Pseudomonas* was a dominant bacteria in nicotine degradation and nicotine served as the unique carbon and nitrogen source for it. Other studies further revealed that nicotine could be degraded by *Pseudomonas*, demonstrating that this process was achieved through the pyrrolidine pathway [28, 29]. Nicotine is the chemical that causes addiction to tobacco products. In addition, nicotine and other chemical compounds produced by the burning of tobacco are primary causes of health harm, such as lung disease [30], respiratory disease [31], and cancer [32]. Ebrahimpour et al. [33] observed that nicotine might downregulate anti-inflammatory microRNAs and stimulate growth factors to accelerate the idiopathic pulmonary fibrosis process. Thus, the focus of reducing tobacco harm should be to decrease the content of nicotine level in tobacco products and change the addictive nature of combustible cigarettes [34].

In the present study, we found a significantly lower content of nicotine in fermentation at 45 °C for 6 weeks than that in the other groups (Table 3). At the same time, the abundance of *Pseudomonas* was correspondingly higher than that under other conditions. Furthermore, sensory evaluation indicated that the sensory qualities of fermentation at 45 °C for 6 weeks were the best among the tested groups (Table 4). Taken together, fermenting at 45 °C for 6 weeks might be the optimal condition of tobacco fermentation, which not only maintained the better quality, but also had low content of nicotine and might eventually reduce harm to humans to some extent. Additionally, *Sphingomonas* had the ability of degrading nicotine, and might be suitable for the reduction of nicotine in tobacco [35, 36]. However, the association between microflora abundance and nicotine content was not explored in this study. Therefore, to reduce the harmful substances in cigarettes, *Pseudomonas* and *Sphingomonas* could be added in the tobacco fermentation, and this is the focus of our further research.

Metabolome analysis showed that sphingolipid metabolism was an essential pathway during tobacco fermentation (Fig. 6). Sphingolipids are considered as modulators of cellular interaction and recognition [37]. The functions of sphingolipids are diverse, including cell death, inflammation, and immune responses. In addition, sphingolipids have been reported to play roles in modulating cancer signaling to control tumor suppression or survival [38], and sphingosine 1-phosphate (S1P) is a bioactive molecule within sphingolipid metabolites [39]. Chen et al. [40] indicated that significant emphysema changes were observed in the pulmonary parenchyma of smoker mice, where the level of S1P was increased in the lungs of cigarette smoke-exposed mice. Meanwhile, sphingomyelinase 2 is activated by smoking and induces the apoptosis of lung cells [41]. These studies suggest that sphingolipids and their metabolism are closely associated with the development and treatment of lung-related diseases caused by smoking. In this study, we found that phytosphingosine might be involved in the sphingolipid metabolism pathway, showing significant increase with the extension of fermentation time. Phytosphingosine participates in diverse cellular processes. Xiong et al. [42] revealed that phytosphingosine level was reduced in the lung tissue of acute lung injury mouse models. Moreover, Park et al. [43] found that phytosphingosine could induce non-small cell lung carcinoma cells apoptosis. In the current study, we found that the level of phytosphingosine was the highest at 6 weeks of fermentation (Supplementary Table 1). Based on the above reports, we speculated that phytosphingosine might alleviate the lung damage caused by harmful substances in smoke to a certain extent. Another metabolite, sphingosine, was also enriched in the sphingolipid metabolism pathway (Supplementary Table 2). Sphingosine is reported to be related to the development of cancer and inflammation [44]. Besides, evidence showed that sphingosine was associated with lung carcinoma signaling pathways which were activated by smoking [45]. Tobacco carcinogen NNK can activate a Raf-1/MAP kinase pathway and stimulate cell proliferation in human small cell lung carcinoma cells, while sphingosine might block this process [46, 47]. Moreover, we found an increase of sphingosine level in fermentation for 6 weeks (Supplementary Table 1). Taken together, these studies further indicated that fermentation at 45 °C for 6 weeks might be the optimum conditions, and phytosphingosine as well as sphingosine might reduce the harm of hazardous substances to the human body via sphingolipid metabolism pathway. However, the specific function of phytosphingosine and sphingosine in tobacco fermentation remains to be further study. Furthermore, the mechanisms of tobacco damage reduction through specific metabolites need further experimental verification.

## Conclusion

In summary, the changes in the microbial community and metabolites during the tobacco fermentation process were systematically studied. Fermentation at 45 °C for 6 weeks was considered to be the optimum fermentation condition due to it maintained favorable sensory qualities of tobacco and reduced the nicotine content to a certain extent. The microorganisms (such as *Pseudomonas* and *Sphingomonas*) and metabolites (such as phytosphingosine and sphingosine) had crucial roles in the tobacco fermentation. Additionally, metabolites might affect the quality of cigarettes through the sphingolipid metabolism pathway. However, the underlying mechanisms of the relationship between microbiota and metabolites warrant further investigation.

## Methods

### Sample collection and tobacco fermentation

Tobacco samples (Shiyan No.1) were grown in Deyang (Sichuan, China). After ripening, the middle leaves were harvested and sun-dried for 5 weeks. Then, the sun-dried tobacco was stacked in the fermentation room. The tobacco leaves were fermented at 25 °C, 35 °C, and 45 °C (90% relative humidity), and samples were collected after 2, 4, and 6 weeks, respectively. Additionally, the sun-dried unfermented tobacco leaves were considered as control group. To ensure the uniformity of the samples, the tobacco leaves were collected from five different parts of the tobacco stack and mixed as samples of each group. The fermentation process was performed in triplicate. Then, the quality and sensory evaluation of fermented tobacco leaves were detected. The measurement was repeated five times.

### Physiological indicators and sensory evaluation

#### Determination of aroma components

The content of aroma components in tobacco leaves was determined using pre-treatment of n-hexane extraction at room temperature improved by Shanghai tobacco technology center and liquid chromatography–gas chromatography (LC-GC) technique [48]. Approximately 0.2 g of powder samples were weighed and put into a 20 mL tube, supplemented with 5 mL extraction solvent (n-hexane: Tert-butyl methyl ether, v:v = 1:1) and 200 μL internal standard solution (α-ionone, 11.2 μg/mL). After standing for 24 h at 25 °C, the solution was vortexed for 1 min and then centrifuged at 3000 rpm for 10 min. Then, 2 mL of the supernatant was extracted and transferred to a chromatographic tube. The LC analysis was performed on an Agilent 1260 system (Agilent Technologies, USA) using a Waters Styragel HR 0.5 column (30 cm × 4.6 cm i.d., 5 m) (Waters Corp., USA). Dichloromethane was used as mobile phase, the flow rate was 0.25 mL/min, the column was maintained at 30 °C, and the injection volume was 20 μL. The diode array detector (DAD) detection wavelengths were 238 nm, 254 nm, and 320 nm. Meanwhile, the LC-GC transfer was performed as described by Qi et al. [48]. The GC analysis was employed on an Agilent 7890A GC system (Agilent Technologies, USA) equipped with electron caputure detector (ECD). Then, GC/MS separation was performed on a DB-5MS capillary column (30 m × 0.25 mm i.d., 0.25 m df) from Agilent Technologies (USA). The oven temperature was maintained at 40 °C for 14 min, then increased to 290 °C at 4 °C/min and maintained for 5 min. The GC/MS transmission line temperature was 280 °C, the MS ion source temperature was 230 °C, quadrupole temperature was 170 °C, and mass ranged from 45 to 350 amu. Chemical components detected in GC-MS analysis were identified using NIST98 and Wiley 6.0 library software.

#### TSNAs, alkaloids, and nitrate assays

TSNAs assays were performed by Shanghai Tobacco Group Beijing Cigarette Factory using online solid-phase extraction (SPE)-LC-MS/MS (SPE-LC: Spark Holland, Symbiosis Pico; MS/MS: AB Sciex triple quad 5500) [49]. Four common TSNAs, including N′-nitrosonornicotine (NNN), N′-nitrosoanatabine (NAT), N′-nitrosoanabasine (NAB), and NNK, were tested. Samples (1.0 g) were transferred into a 50 mL conical flask. Then, four deuterated internal standard solutions (40 μL) and 30 mL ammonium acetate solution (100 mmol/L) were added. The sample was shaken and extracted at 25 °C for 60 min (200 r/min) and then filtered with a 0.45 μm Water phase filter membrane. After collecting the filtrate, LC-MS/MS was used to detect the content of NNN, NAT, NAB, and NNK. The sum of the four TSNAs represented the total amount of TSNAs.

Alkaloids detection was conducted based on previously published methods [50]. Briefly, 200 mg of the sample was weighed and put into a 10 mL glass bottle. Next, 1.5 mL 10% NaOH solution and 3 mL methyl tert-butyl ether were added. The mixed solution was shaken for 5 min and kept standing for 24 h. Then, the supernatant was extracted and passed through gas chromatography-Hydrogen flame ionization detector (Agilent 7890A, Agilent Technologies, USA) to detect four alkaloids, including nicotine, nornicotine, neonicotine, and anatabine.

The nitrate content was detected following the method described by Da et al. [50]. For that, 400 mg of cigarette sample in a triangular flask was re-suspended in 50 mL 1% CH_3_COOH solution and shaken for 20 min. After NO_3_- was extracted and filtered, the activated carbon was supplemented for decolorization and the solution was filtered. After that, 15 mg of zinc powder was added and the sample was subjected to a slight oscillation for 15 min. Two mL nitrate reagent was added to the 8 mL of the intermediate filtrate, and let standing for 15 min. The absorbance was determined on a 721 spectrophotometer at a wavelength of 540 nm using a blank reagent as a reference.

#### Sensory quality evaluation

Samples were cut into rolls to make cigarettes, and then evaluated by seven experts from the Technology Center of Henan China Tobacco Industry Co., Ltd. and the Tobacco College of Henan Agricultural University. Sensory quality was calculated according to the standard of the reference YC/T138–1998 of China [11]. Specifically, this measure contained 9 items rated on a nine-point scale, ranging from 0 to 9 score. The quality indexes contained aroma quality, aroma quantity, smoke density, offensive odor, physiological strength, irritation, aftertaste, combustibility, and cigarette ash. Furthermore, the result of each index was the average of seven people’s scores.

### Metabolomics analysis

#### Sample preparation and LC-MS analysis

The quality detection and sensory analysis showed that 45 °C was the optimal temperature for the fermentation process. Thus, we selected tobacco leaves fermented at 45 °C for 2 weeks, 4 weeks, and 6 weeks for metabolomics analysis. The sun-dried unfermented tobacco leaves served as control. The groups were designated as group 1 (control), group 2 (2 weeks), group 3 (4 weeks), and group 4 (6 weeks), with five samples in each group.

Metabolomics analysis was performed using LC-MS. In brief, 0.2 g tobacco powder of each sample was weighed and put into 20 mL tubes. The metabolites were extracted by adding 5 mL n-hexane:tert-butyl methyl ether solution (v:v, 1:1) and 200 μL α-Ionone (11.2 μg/mL) internal standard solution. After standing for 24 h at room temperature, the samples were vortexed at 200 rpm for 1 min. The mixture was centrifuged at 2000 rpm for 10 min. Finally, 2 mL supernatant was placed in an LC-MS sample bottle for detecting. The method of LC-MS analysis was consistent with the detection of aroma components as described above.

#### Data pre-processing and analysis

The raw data obtained from LC-MS were pre-processed using the Progenesis QI (Waters Corporation) software, including missing value recoding and normalization. Then, a data matrix was obtained and converted using the SIMCA-P 14.1 software (Umetrics, Umea, Sweden). According to the expression of metabolites, the PCA was performed to evaluate the similarity of samples within groups and the difference of samples among groups. Moreover, to investigate the biological functions of metabolites, the KEGG [4] and HMDB 4.0 [5] databases were applied to perform identification and annotation.

#### Identification of the differential metabolites

To obtain the differential metabolites between different groups (group 2 vs. group 1, group 3 vs. group 1, and group 4 vs. group 1), the multiple statistical methods and significance difference test were performed. A series of statistical analyses, including PCA, supervised partial least-squares discriminant analysis (PLS-DA), and orthogonal partial least-squares discriminant analysis (OPLS-DA), were applied. Differential metabolites were identified based on the statistically significant threshold of VIP values obtained from the OPLS-DA model and *p*-value from Student’s *t*-test on the normalized peak areas. Metabolites with VIP > 1 and *p*-value < 0.05 were considered statistically significant. Meanwhile, the ellipse in the plots showed the Hotelling’s T2 confidence region, which defined the 95% confidence interval (CI) of the modeled variation. The PLS-DA and OPLS-DA models were verified using a permutation test repeated 200 times.

#### Metabolic pathway enrichment analyses

To observe the distribution of metabolites in each group, a Venn diagram was generated. Moreover, the metabolite information in the pathways was extracted based on the KEGG database, and the pathway of differential metabolites was obtained using the hypergeometric test method. The *p*-value was corrected by using the Benjamini-Hochberg (BH) method, and *p*-value < 0.05 was set as the cut-off criteria.

### 16 s rDNA/rRNA amplicon sequencing and analysis

#### DNA extraction from the tobacco leaves and sequencing approach

In order to investigate the changes of microbiota during the fermentation process, 16 s rRNA gene sequencing was also performed for these four groups, with each group included five samples. Ten grams of tobacco leaves of each sample were collected and placed in a flask with 250 mL sterilized 0.1 M phosphate buffer (pH 7.0) for 30 min. Later, the tobacco leaves were washed with a sonicator for 10 min and the microorganisms were collected by centrifugation at 10,000×g for 30 min. Total microbial genomic DNA was extracted using an ExPro Tobacco DNA Kit (Gene Answer) based on the guides provided by the manufacturer. The genomic DNA isolated from samples was used as template for 16S rRNA gene amplification. The V3-V4 regions of the 16 s rRNA gene (from 338F to 806R) were amplified from DNA using previously published primers [51]. PCR amplification was conducted using TransStart® Fastpfu DNA Polymerase kit on the ABI GeneAmp® 9700 (Applied Biosystems) and each sample was assayed in triplicate. Then, PCR products were detected using 2% agarose gel electrophoresis and recovered using an AxyPrepDNA Gel Recovery Kit (Axygen Bioscience), followed by elution with Tris-HCl. Then, the PCR products were quantified using QuantFlour™-ST Blue Fluorescence Quantitative System (Promega) and the amplicon was sequenced using Illumina MiSeq platform. The raw sequencing files of twenty tobacco samples were uploaded in the NCBI SRA database (http://www.ncbi.nlm.nih.gov/bioproject/660877) under the accession number of PRJNA660877.

#### Bioinformatics analysis

The raw sequencing data were de-multiplexed and quality controlled by FLASH Trimmomatic software [52]. The high-quality sequencing reads were analyzed and clustered into OTU using Usearch (version: 7.0, http://drive5.com/usearch/) with a 97% similarity threshold. Additionally, taxonomic assignments of OTUs were conducted using the QIIME (version: 1.9.0, http://qiime.org/scripts/assign_taxonomy.html) software through comparison with RDP classifier (version: 2.2, http://sourceforge.net/projects/rdp-classifier/) and Silva database (release 128, http://www.arb-silva.de). Based on the OTUs table, the alpha-diversity analysis was performed, which evaluated the species diversity of samples via calculating Ace, Chao, Shannon, and Simpson indexes. The *t*-test was used to assess the significant difference between groups, and results were visualized using the R software (version: 2.15.3, http://www.R-project.org). Besides, the rarefaction curve was analyzed using the Mothur (version: 1.39.5, https://mothur.org/). To further analyze the species composition, the Venn diagram was used to count the number of the common and unique OTUs in multiple samples, and the pie-chart was utilized to show the relative abundance of each microbiota in the samples. The beta-diversity analysis was aimed to examine the similarity of community structure among groups. In this study, beta-diversity results were visualized using PCA, PCoA, and NMDS plots. Differential OTUs were identified using analysis of variance (ANOVA), and *p*-value < 0.05 was defined as statistically significant. In addition, the phylogenetic investigation of communities by reconstruction of unobserved states program (PICRUSt) was applied to predict the function of microbiota and *p*-value less than 0.05 was set as the threshold of significant enrichment.

### Integration of amplicon sequencing and metabolomics analysis

For studying the relationship between microbiota and metabolites, an integration analysis was performed. According to the results of KEGG pathways analysis, the same or similar pathways, which existed both in 16 s rRNA gene sequencing and metabolome analysis, were selected.

### Statistical analysis

One-way ANOVA with Bonferroni’s multiple comparisons test and Student’s *t*-test were performed using SPSS 17.0 software (IBM, USA). The significance threshold was set at *p*-value < 0.05.

## Supplementary Information

**Additional file 1:** **Supplementary Figure S1.** The primary physicochemical indexes of fermented tobacco leaves. A: NNN; B: TSNAs; C: Alkaloids; D: Nitrates. NNN: N′-nitrosonornicotine; TSNAs: Tabacco-specific Nitrosamines. Error bars represent standard errors.

**Additional file 2:** **Supplementary Figure S2.** Score scatter plot of the PCA model. A: POS mode; B: NEG mode. PCA: principal component analysis; POS: positive; NEG: negative. Group 1 represents the sun-dried unfermented tobacco leaves; group 2 represents leaves fermented at 45 °C for 2 weeks; group 3 represents leaves fermented at 45 °C for 4 weeks; and group 4 represents leaves fermented at 45 °C for 6 weeks; QC represents quality control group.

**Additional file 3:** **Supplementary Figure S3.** PCA model score scatter plot, PLS-DA model, and OPLS-DA model for the different groups. A: PCA model from NEG; B: PCA model from POS; C: PLS-DA model from NEG; D: PLS-DA model from POS; E: OPLS-DA model from NEG; F: OPLS-DA model from POS. PLS-DA: partial least squares-discriminant analysis; OPLS-DA: orthogonal partial least squares-discrimination analysis.

**Additional file 4:** **Supplementary Figure S4.** The rarefaction curves for each sample. A: Shannon value; B: Sobs value.

**Additional file 5:** **Supplementary Figure S5.** The beta diversity comparison among four groups. A: PCA plot; B: PCoA plot; C: NMDS plot. PCoA: principal-coordinate analysis; NMDS: non-metric multidimensional scaling.

**Additional file 6:** **Supplementary Table S1.** The detail information of annotated metabolites. (XLS 393 kb)

**Additional file 7:** **Supplementary Table S2.** Top 20 KEGG pathway and enriched metabolites. **Supplementary Table S3.** Top 20 KEGG pathways of differential OTUs.

## Data Availability

The dataset generated or analyzed and strain used in the current study were submitted to NCBI SRA database with the accession no. PRJNA660877.

## Abbreviations

RH: Relative humidity
TSNAs: Tobacco-specific nitrosamines
LC-MS: Liquid chromatography-mass spectrometry
PCA: Principal component analysis
POS: Positive
NEG: Negative
KEGG: Kyoto Encyclopedia of Genes and Genomes
HMDB: Human Metabolome Database
VIP: Variable importance in the projection
OTUs: Operational taxonomic units
PCoA: Principal coordinate analysis
NMDS: Non-metric multidimensional scaling
S1P: Sphingosine 1-phosphate
LC-GC: Liquid chromatography–gas chromatography
ECD: Electron caputure detector
SPE: Solid-phase extraction
NNN: N′-nitrosonornicotine
NAT: N′-nitrosoanatabine
NAB: N′-nitrosoanabasine
PLS-DA: Partial least-squares discriminant analysis
OPLS-DA: Orthogonal partial least-squares discriminant analysis
CI: Confidence interval
BH: Benjamini-Hochberg
