# Supplementary figures and images for: Influence of microbiota and metabolites on the quality of tobacco during fermentation
Source: BMC Microbiol. 2020 Nov 19;20:356. doi: 10.1186/s12866-020-02035-8 (PMC7678276; doi:10.1186/s12866-020-02035-8)

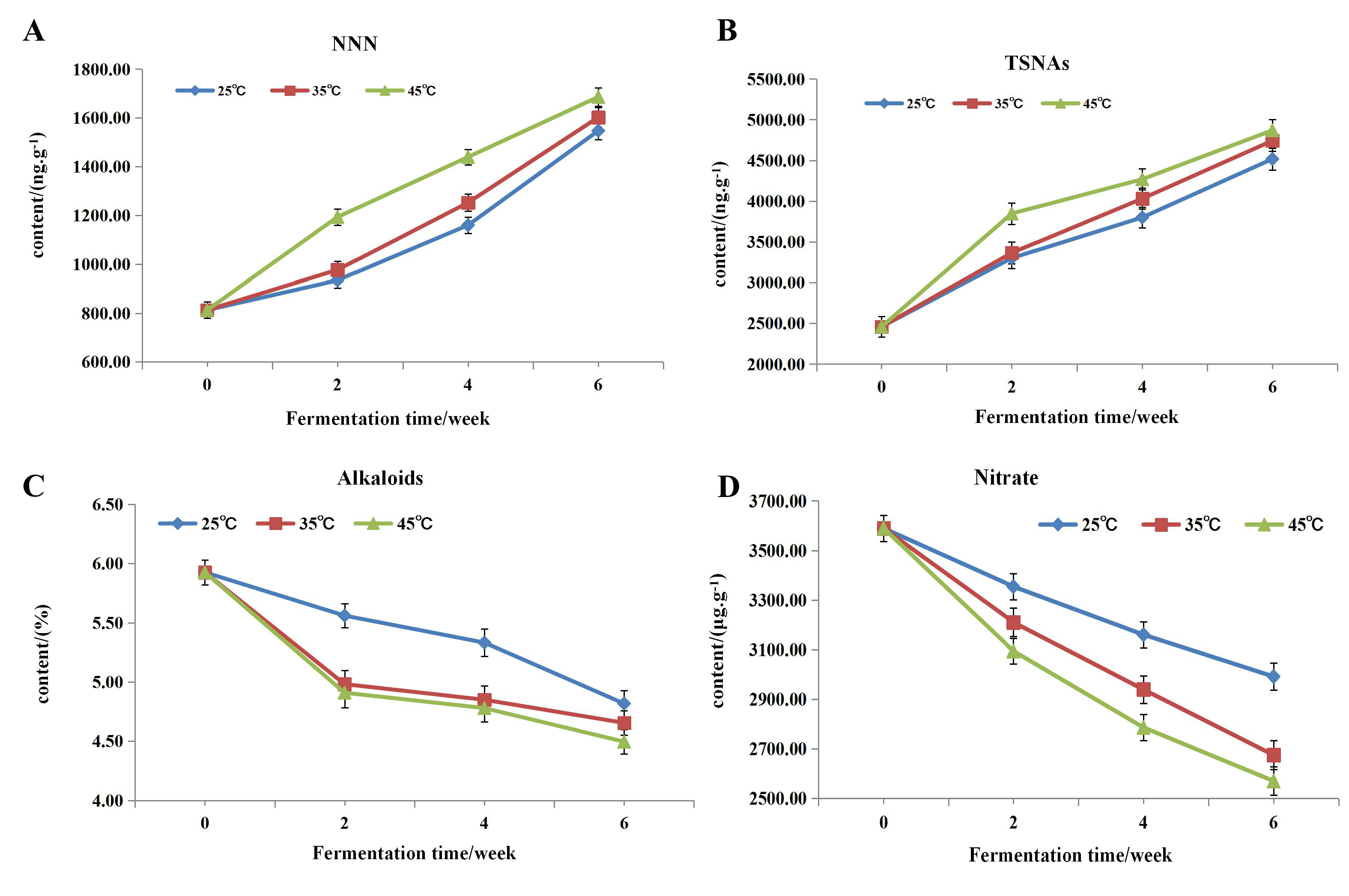

Supplement: Supplementary file 1 — Additional file 1: Supplementary Figure S1. The primary physicochemical indexes of fermented tobacco leaves. A: NNN; B: TSNAs; C: Alkaloids; D: Nitrates. NNN: N′-nitrosonornicotine; TSNAs: Tabacco-specific Nitrosamines. Error bars represent standard errors. [file 12866_2020_2035_MOESM1_ESM.tif]

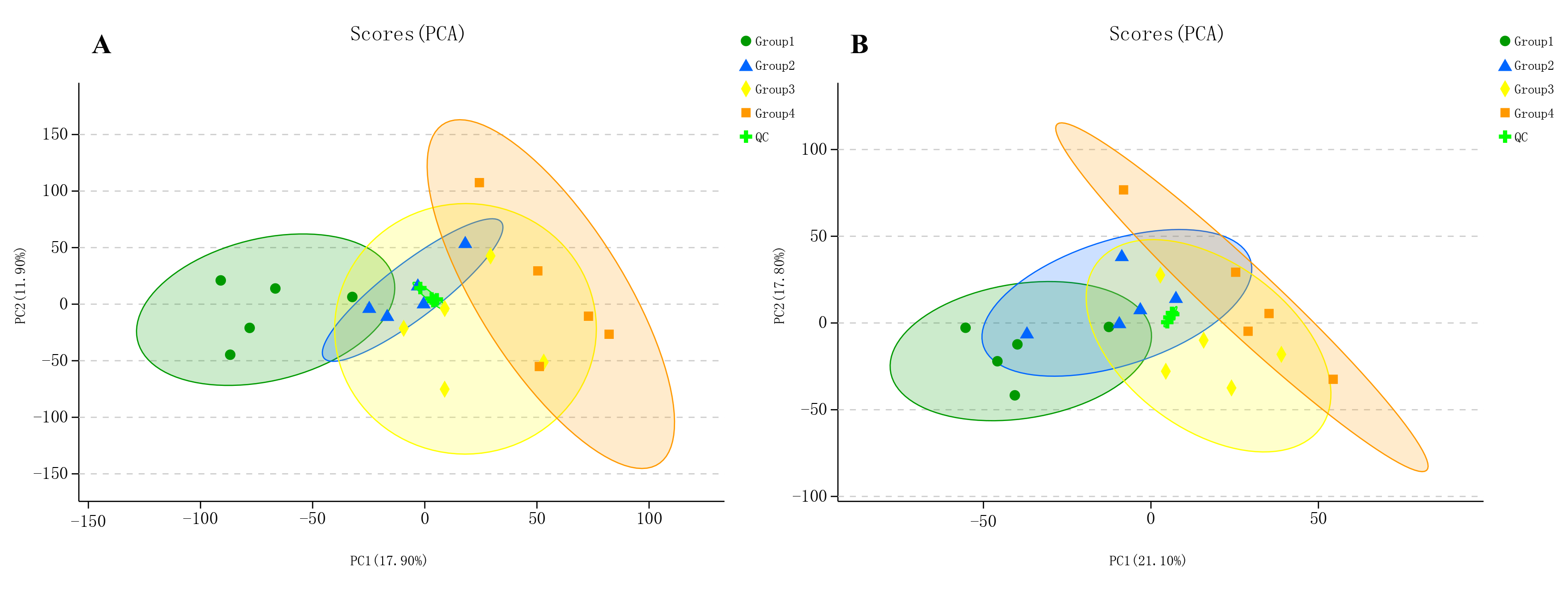

Supplement: Supplementary file 2 — Additional file 2: Supplementary Figure S2. Score scatter plot of the PCA model. A: POS mode; B: NEG mode. PCA: principal component analysis; POS: positive; NEG: negative. Group 1 represents the sun-dried unfermented tobacco leaves; group 2 represents leaves fermented at 45 °C for 2 weeks; group 3 represents leaves fermented at 45 °C for 4 weeks; and group 4 represents leaves fermented at 45 °C for 6 weeks; QC represents quality control group. [file 12866_2020_2035_MOESM2_ESM.tif]

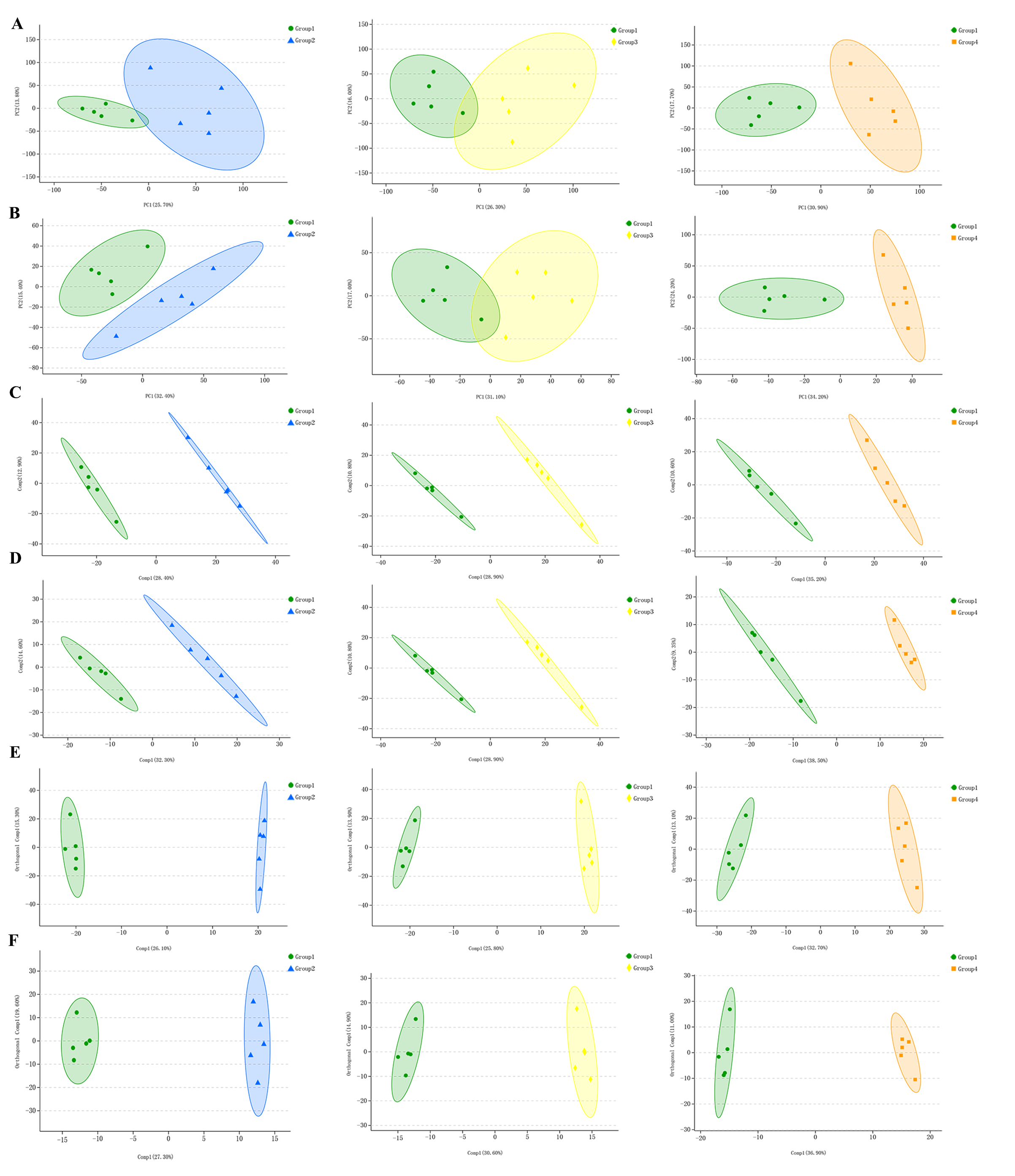

Supplement: Supplementary file 3 — Additional file 3: Supplementary Figure S3. PCA model score scatter plot, PLS-DA model, and OPLS-DA model for the different groups. A: PCA model from NEG; B: PCA model from POS; C: PLS-DA model from NEG; D: PLS-DA model from POS; E: OPLS-DA model from NEG; F: OPLS-DA model from POS. PLS-DA: partial least squares-discriminant analysis; OPLS-DA: orthogonal partial least squares-discrimination analysis. [file 12866_2020_2035_MOESM3_ESM.tif]

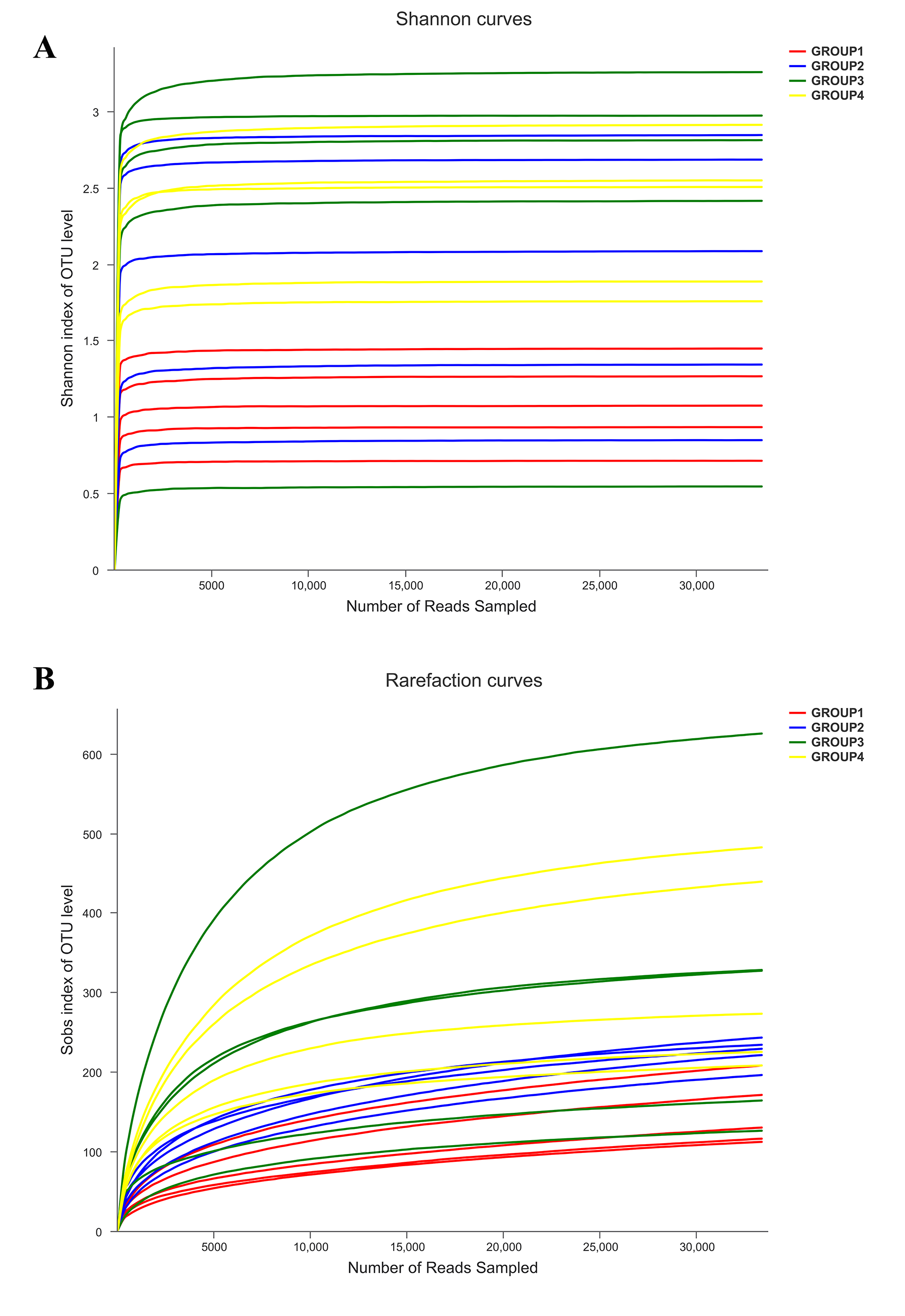

Supplement: Supplementary file 4 — Additional file 4: Supplementary Figure S4. The rarefaction curves for each sample. A: Shannon value; B: Sobs value. [file 12866_2020_2035_MOESM4_ESM.tif]

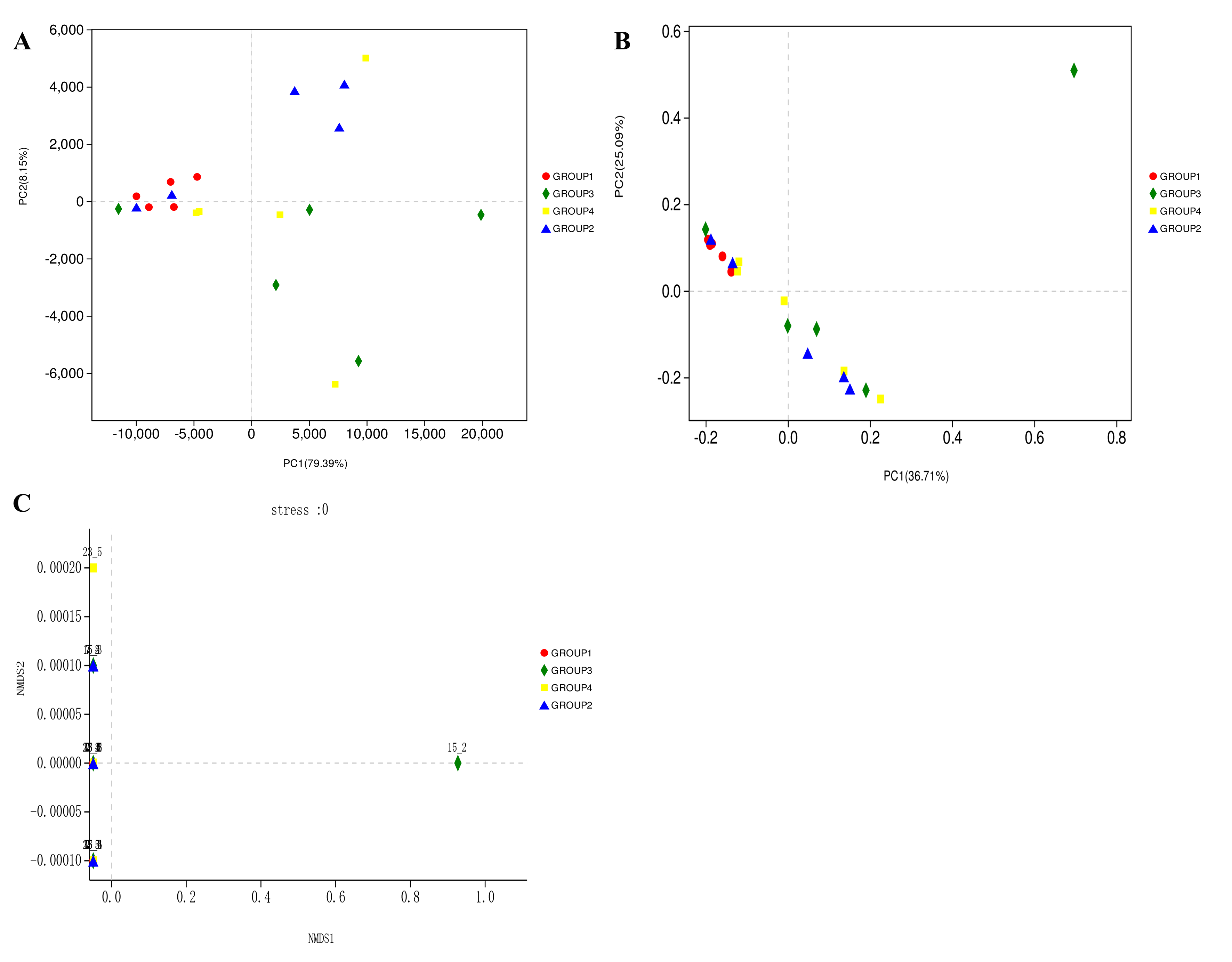

Supplement: Supplementary file 5 — Additional file 5: Supplementary Figure S5. The beta diversity comparison among four groups. A: PCA plot; B: PCoA plot; C: NMDS plot. PCoA: principal-coordinate analysis; NMDS: non-metric multidimensional scaling. [file 12866_2020_2035_MOESM5_ESM.tif]
