# Supplementary material for: Influence of microbiota and metabolites on the quality of tobacco during fermentation
Source: BMC Microbiol. 2020 Nov 19;20:356. doi: 10.1186/s12866-020-02035-8 (PMC7678276; doi:10.1186/s12866-020-02035-8)
Supplement: Supplementary file 7 — Additional file 7: Supplementary Table S2. Top 20 KEGG pathway and enriched metabolites. Supplementary Table S3. Top 20 KEGG pathways of differential OTUs. [file 12866_2020_2035_MOESM7_ESM.doc]

**Supplementary Table 2. Top 20 KEGG pathway and enriched metabolites**

| **Pathway** | **First_category** | **Second_category** | **Description** | **Count** | **Metabolite** |
| --- | --- | --- | --- | --- | --- |
| map01100 | Metabolism | Global and overview maps | Metabolic pathways | 21 | Gamma-Linolenic acid;Inosine;L-Phenylalanine;Cholesterol glucuronide;N-Acetylglutamic acid;Phytosphingosine;L-Histidine;Adenine;3-ketosphinganine;Gamma-Aminobutyric acid;Rutin;(+)-Abscisic Acid;Betaine;Quercetin 3-O-glucoside;Sphingosine;D-Proline;Thymidine;L-Tryptophan;SM(d18:0/22:3(10Z,13Z,16Z));Kaempferol;Caffeine |
| map01110 | Metabolism | Global and overview maps | Biosynthesis of secondary metabolites | 13 | L-Tryptophan;L-Phenylalanine;N-Acetylglutamic acid;L-Histidine;2,3-Dihydroxybenzoic acid;Astragalin;Rutin;(+)-Abscisic Acid;Methyl jasmonate;Quercetin 3-O-glucoside;Anatabine;Kaempferol;Caffeine |
| map01130 | Metabolism | Global and overview maps | Biosynthesis of antibiotics | 5 | L-Tryptophan;L-Phenylalanine;Novobiocin;2,3-Dihydroxybenzoic acid;N-Acetylglutamic acid |
| map01060 | Metabolism | Chemical structure transformation maps | Biosynthesis of plant secondary metabolites | 5 | L-Histidine;(+)-Abscisic Acid;L-Tryptophan;L-Phenylalanine;Caffeine |
| map00600 | Metabolism | Lipid metabolism | Sphingolipid metabolism | 4 | Phytosphingosine;Sphingosine;3-ketosphinganine;SM(d18:0/22:3(10Z,13Z,16Z)) |
| map01230 | Metabolism | Global and overview maps | Biosynthesis of amino acids | 4 | L-Histidine;L-Phenylalanine;L-Tryptophan;N-Acetylglutamic acid |
| map01061 | Metabolism | Chemical structure transformation maps | Biosynthesis of phenylpropanoids | 4 | L-Tryptophan;L-Phenylalanine;2,3-Dihydroxybenzoic acid;Kaempferol |
| map00944 | Metabolism | Biosynthesis of other secondary metabolites | Flavone and flavonol biosynthesis | 4 | Rutin;Astragalin;Kaempferol;Quercetin 3-O-glucoside |
| map01040 | Metabolism | Lipid metabolism | Biosynthesis of unsaturated fatty acids | 3 | 11,14,17-Eicosatrienoic acid;Eicosapentaenoic Acid;Gamma-Linolenic acid |
| map04974 | Organismal Systems | Digestive system | Protein digestion and absorption | 3 | L-Tryptophan;L-Phenylalanine;L-Histidine |
| map02010 | Environmental Information Processing | Membrane transport | ABC transporters | 3 | L-Histidine;L-Phenylalanine;Betaine |
| map01210 | Metabolism | Global and overview maps | 2-Oxocarboxylic acid metabolism | 3 | L-Tryptophan;L-Phenylalanine;N-Acetylglutamic acid |
| map01120 | Metabolism | Global and overview maps | Microbial metabolism in diverse environments | 3 | 2,3-Dihydroxybenzoic acid;Gamma-Aminobutyric acid;Caffeine |
| map01070 | Metabolism | Chemical structure transformation maps | Biosynthesis of plant hormones | 3 | (+)-Abscisic Acid;L-Tryptophan;L-Phenylalanine |
| map01063 | Metabolism | Chemical structure transformation maps | Biosynthesis of alkaloids derived from shikimate pathway | 3 | L-Tryptophan;L-Phenylalanine;3,4-Dihydroxybenzaldehyde |
| map00970 | Genetic Information Processing | Translation | Aminoacyl-tRNA biosynthesis | 3 | L-Tryptophan;L-Phenylalanine;L-Histidine |
| map05230 | Human Diseases | Cancers: Overview | Central carbon metabolism in cancer | 3 | L-Tryptophan;L-Phenylalanine;L-Histidine |
| map00360 | Metabolism | Amino acid metabolism | Phenylalanine metabolism | 3 | Phenylethylamine;L-Phenylalanine;2-Phenylethanol |
| map00410 | Metabolism | Metabolism of other amino acids | beta-Alanine metabolism | 2 | L-Histidine;Gamma-Aminobutyric acid |
| map00966 | Metabolism | Biosynthesis of other secondary metabolites | Glucosinolate biosynthesis | 2 | L-Tryptophan;L-Phenylalanine |

**Supplementary Table 3.** Top 20 KEGG pathways of differential OTUs

| **Pathway** | ***P*-value** | **FDR** | **Description** |
| --- | --- | --- | --- |
| ko01051 | 0.007695272 | 0.312310776 | Biosynthesis of ansamycins |
| ko01053 | 0.008576306 | 0.312310776 | Biosynthesis of siderophore group nonribosomal peptides |
| ko00511 | 0.012730485 | 0.312310776 | Other glycan degradation |
| ko03015 | 0.012768713 | 0.312310776 | mRNA surveillance pathway |
| ko02010 | 0.024092654 | 0.312310776 | ABC transporters |
| ko00943 | 0.024335714 | 0.312310776 | Isoflavonoid biosynthesis |
| ko00040 | 0.025449114 | 0.312310776 | Pentose and glucuronate interconversions |
| ko04145 | 0.025552468 | 0.312310776 | Phagosome |
| ko00562 | 0.036733918 | 0.312310776 | Inositol phosphate metabolism |
| ko00600 | 0.037138644 | 0.312310776 | Sphingolipid metabolism |
| ko00030 | 0.039751202 | 0.312310776 | Pentose phosphate pathway |
| ko04011 | 0.04572739 | 0.312310776 | MAPK signaling pathway - yeast |
| ko00120 | 0.046958741 | 0.312310776 | Primary bile acid biosynthesis |
| ko00940 | 0.047150494 | 0.312310776 | Phenylpropanoid biosynthesis |
| ko00590 | 0.047644781 | 0.312310776 | Arachidonic acid metabolism |
| ko04622 | 0.048497855 | 0.312310776 | RIG-I-like receptor signaling pathway |
| ko04976 | 0.0500098 | 0.312310776 | Bile secretion |
| ko00053 | 0.050806179 | 0.312310776 | Ascorbate and aldarate metabolism |
| ko00351 | 0.051936284 | 0.312310776 | 1,1,1-Trichloro-2,2-bis(4-chlorophenyl)ethane (DDT) degradation |
| ko00460 | 0.054647333 | 0.312310776 | Cyanoamino acid metabolism |
